# Clinical insights into the three-dimensional anatomy of cheek teeth in alpacas based on micro-computed tomography - Part 2: Maxillary cheek teeth

**DOI:** 10.1186/s12917-021-03039-w

**Published:** 2022-01-03

**Authors:** Kirsten Proost, Matthieu N. Boone, Ivàn Josipovic, Bart Pardon, Koen Chiers, Lieven Vlaminck

**Affiliations:** 1 Department of Surgery and Anesthesiology of Domestic Animals, Faculty of Veterinary Medicine, Ghent University, Merelbeke, Belgium; 2 Department of Physics and Astronomy – Radiation Physics, Faculty of Science, RP-UGCT, Ghent University, Ghent, Belgium; 3 Department of Large Animal Internal Medicine, Faculty of Veterinary Medicine, Ghent University, Merelbeke, Belgium; 4 Department of Pathology, Bacteriology and Poultry Diseases, Faculty of Veterinary Medicine, Ghent University, Merelbeke, Belgium

**Keywords:** Alpaca dentistry, Apical infection, Common pulp chamber, Dental anatomy, Dental disease, Dental pathology, New world camelids, Sub-occlusal dentinal thickness, Tooth root abscess

## Abstract

**Background:**

Scientific knowledge regarding alpaca dentistry is relatively limited despite its clinical implications. The present gap in available supportive data leads to limited treatment options for dental pathology in alpacas in comparison to other species. The main goal of this study was to gain novel insights into the general and pulp morphology of maxillary cheek teeth to allow development of more advanced treatment strategies in the future. Also, the risk of causing pulp exposure when floating maxillary cheek teeth was of particular interest. Concurent research focusing on the anatomy of mandibular cheek teeth has been performed accordingly. The results obtained in mandibular teeth are expected to be non-extrapolatable because of the structural differences between mandibular and maxillary teeth.

**Results:**

Pulp morphology of maxillary cheek teeth showed great variation. A common pulp chamber was identified in 46/83 (55.4%) teeth with a mean dental age of 2 years and 7 months (± 2 years and 5 months). Pulpal segmentation was more commonly observed in teeth of increasing age. Full columnar segmentation was seen in 33/69 teeth (47.8%), whereas within-column segmentation was observed in 36/83 teeth (43.4%). Age and degree of segmentation of the pulpal tissue varied greatly according to Triadan position. Physical contact between roots of adjacent teeth was found in the majority of examined molars (range 82–94%) which resulted in morphological adaptations at the level of the root tips. The measured sub-occlusal dentinal thickness was as low as 0.46 mm above pulp horn 2 in a 14 years and 11 months old Triadan 09, emphasizing the risk of pulp exposure attributed to dental floating.

**Conclusion:**

This study offers an objective description of age-dependent maxillary cheek teeth pulp morphology in alpacas. Current findings are of great value to provide a basis for the development of tooth-saving techniques as a treatment for dental disease in this species. Observed physical contact between the roots of different examined molars may be a facilitating factor in the spread of apical infection in chronically diseased cases. Finally, a conservative approach regarding dental floating is recommended in order to avoid iatrogenic damage to pulp tissue.

## Background

Dental problems are increasingly recognized as a common pathology in the domesticated alpaca population. The early, specific veterinary literature on dental disease in camelids mainly focused on tooth root abscesses [1–3]. Recent studies have shown a high prevalence of other dental problems that are believed to precede tooth root abscesses, indicating that dental disease in general remains underdiagnosed in this species [3, 4]. A lack of sufficient in-depth scientific knowledge with regards to normal anatomy and physiology of alpaca cheek teeth hinders proper understanding of the pathogenesis of specific dental problems [5, 6]. Both detailed information on external anatomical conformation as well as internal pulp characteristics of alpaca cheek teeth are of particular interest in understanding dental pathology and are necessary for the development of efficient advanced treatment strategies. Given the low number of cheek teeth contributing to the grinding surface, development of tooth saving techniques is of particular intrest in this species. In this way, endodontics can provide means to treat teeth diagnosed with pulpitis without the presence of severe destruction in the apical region. Dental floating in alpacas is currently not advised nor performed as a routine procedure like in horses [1, 7]. Incidental wear abnormalities can however necessitate corrective odontoplasty of overgrown teeth [3]. The authors have demonstrated a limited sub-occlusal dentinal thickness (SODT) in alpaca mandibular cheek teeth, predisposing to inadvertent opening of pulp canals or possible irreversible thermal damage of vital pulp tissue which might lead to wear-induced pulp exposure even several months to years post-treatment [7–9]. In aforementioned micro-computed tomography (μ-CT) study on alpaca mandibular cheek teeth detailed insights into general anatomy, specific pulp morphology and SODT are provided [8]. However, these results cannot be simply extrapolated to the maxillary dental arcades as has been demonstrated in different other species such as equids, where gross morphological differences between opposing cheek teeth arcades have been observed [5, 10]. Additionally, maxillary cheek teeth in other species have shown a higher variability in pulp configurations [5, 11].

The present study aimed to obtain more detailed insights into alpaca maxillary cheek tooth anatomy, tooth-specific pulp system morphology, and their age-related changes, including variations of SODT.

## Methods

### Specimen and study design

A cross-sectional study design was used. Using an oscillating saw, 23 hemimaxillae were isolated from the heads of 18 Huacaya alpacas, providing 13 deciduous premolars, 32 permanent premolars and 59 molars. Study animals included 11 female, 4 male and 3 male castrated alpacas. The age of the studied animals ranged from 5 months and 22 days to 15 years and 6 months, with a mean of 6 years and 17 days ± SD 4 years 2 months and 20 days). None of the studied animals died or was euthanased related to dental pathology. All cheek teeth present in selected arcades were used to study prevalence, general anatomy and physical contact between roots of adjacent teeth. Cheek teeth were excluded for further analysis of the SODT and evaluation of normal pulp morphology in the presence of wear abnormalities, extensive periodontal disease, apical infection and occlusal pulp exposure. Only a subset of maxillary cheek teeth could be withheld, comprising of 13 deciduous premolars, 28 permanent premolar and 42 molar cheek teeth. Sample size was primarily determined by the availability of suitable samples. The power of this sample size allowed detection of a difference of 1.3 mm in mean SODT between two different dentition types if an estimated standard deviation of 1.0 mm was taken into account and if 10 teeth per test group were available (mean SODT permanent premolars 4.2 mm - mean SODT molars 2.9 mm). Individual teeth were numbered using the modified Triadan system [12]. The specific dental age was calculated for each maxillary cheek tooth (Table 1) to account for staggered eruption times of specific Triadan positions [13].

**Table 1:** Age distribution of studied maxillary cheek teeth

	Triadan	# Teeth	Mean standard eruption age	Calculated range DA		Mean DA ± sd
				Youngest tooth	Oldest tooth	
Deciduous	06	1	0d	2y2m3d	2y2m3d	/
	07	6	0d	5m22d	3y	1y9m ± 11 m
	08	6	0d	5m22d	3y	1y9m ± 1y
Permanent	07	13	4y2m15d	2d	11y3m15d	4y ± 3y
	08	15	4y2m15d	2d	11y3m15d	4y ± 3y3m
	09	16	7m15d	0d	14y10m15d	5y9m ± 4y5m
	10	14	1y8m15d	0y3m15d	13y9m15d	4y9m ± 3y9m
	11	12	3y2m15d	1y0m2d	12y3m16d	4y9m ± 3y6m

The mean standard eruption age is based on the reported range of eruption times for each specific tooth [13]. The dental age (DA) is calculated by using the animal’s age and the mean standard eruption age. DA is expressed in years (y), months (m) and days (d)

### Scanning parameters

High resolution μ-CT scanning of complete maxillary arcades was performed using the custom-build scanner system HECTOR at the Ghent University Center for X-ray Tomography [14], as previously described for the mandibular arcade [8]. The projection data was reconstructed into a 3D volume using the implementation of the FDK algorithm in the in-house developed software package Octopus Reconstruction. A commercial 3D-rendering software package (VGStudioMAX and myVGL, Volume Graphics GmbH,Germany) was used to allow reconstruction of the maxillary arcade and pulp system of studied cheek teeth and to perform specific measurements.

### Constructing reference planes

**Fig. 1:**
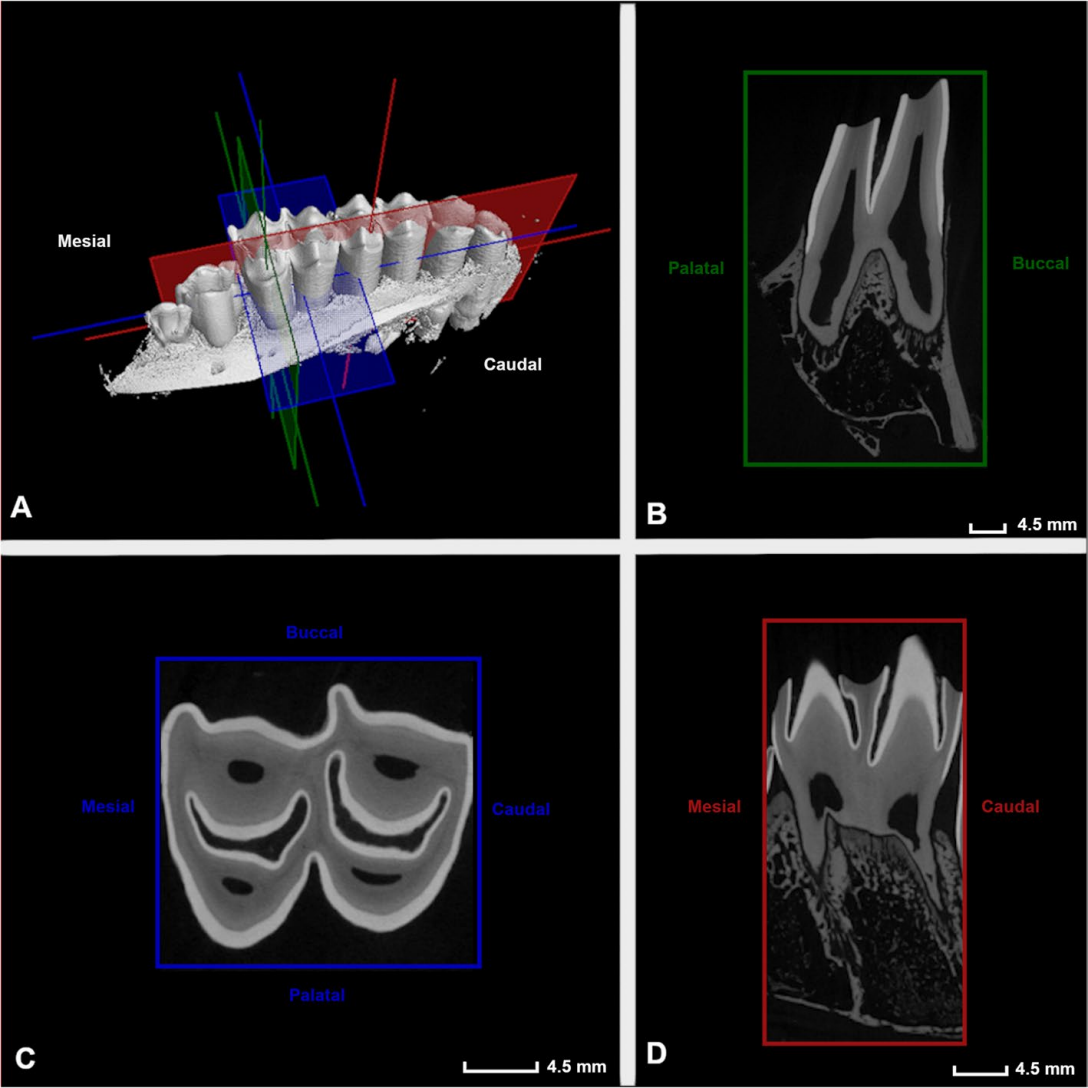
Constructed reference planes. Visual representation of the three constructed reference planes used for the interpretation and study of anatomical aspects of included maxillary cheek teeth, illustrated on the mesial column of a Triadan 109 (4 years and 3 months old alpaca)

To allow for standardized measurements and descriptive characterization of dental structures, three reference planes were drawn for each individual columnar structure in every cheek tooth (Fig. 1).

A first reference plane (B, green) was set, aligned with the most apical enamel point of a specific column and extending according to the longitudinal axis of this specific column. The second refence plane (C, blue) was set perpendicular to the longitudinal axis of the specific studied column. Finally, the third reference plane (D, red) was set in a mesio-distal plane and aligned with the infundibula, if present. A 90° angle was set between all constructed reference planes.

### Descriptive morphology and measurements

**Fig. 2:**
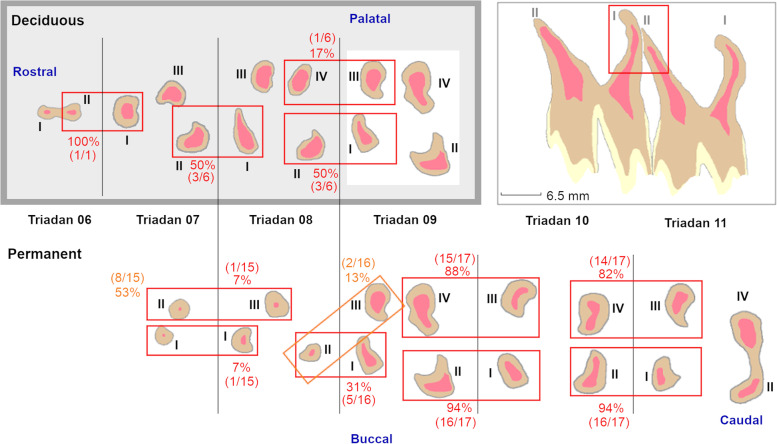
Root canal numbering system of alpaca maxillary cheek teeth. Deciduous teeth are displayed in the grey box (upper left corner). Transverse slices presenting all different root canals at the level of specific Triadan positions are displayed. Pulpal tissue, dentin, enamel and cement are represented in pink, light brown, light yellow and grey, respectively. Root canals are numbered using Roman numerals (I to IV). An example of physical root contact is illustrated in the red box (upper right corner) on a longitudinal section of Triadans 110 and 111 (5-years old animal). The prevalence of root contact is expressed as a percentage adjacent to corresponding red or orange boxes

Two- and three-dimensional findings were used to allow evaluation of general and pulpal tooth morphology. A differentiation was made between crown and roots at the level of the furcation, with the roots located most apically. At crown level, different (pre)molars showed a prominent but localised infolding of the peripheral enamel which divided these teeth in 2 vertical columns [8]. Infundibula were defined as funnel shaped invaginations or cups of enamel from the occlusal surface down [15]. Pulp horns were defined as occlusal extensions originating from centrally located pulpal coalescences. Pulp tissue extending in an apical direction from these coalescences was termed root canal [5, 16]. A root canal numbering system for maxillary cheek teeth, adapted from the numbering system described in horses, is illustrated in Fig. 2 [5].

**Fig. 3:**
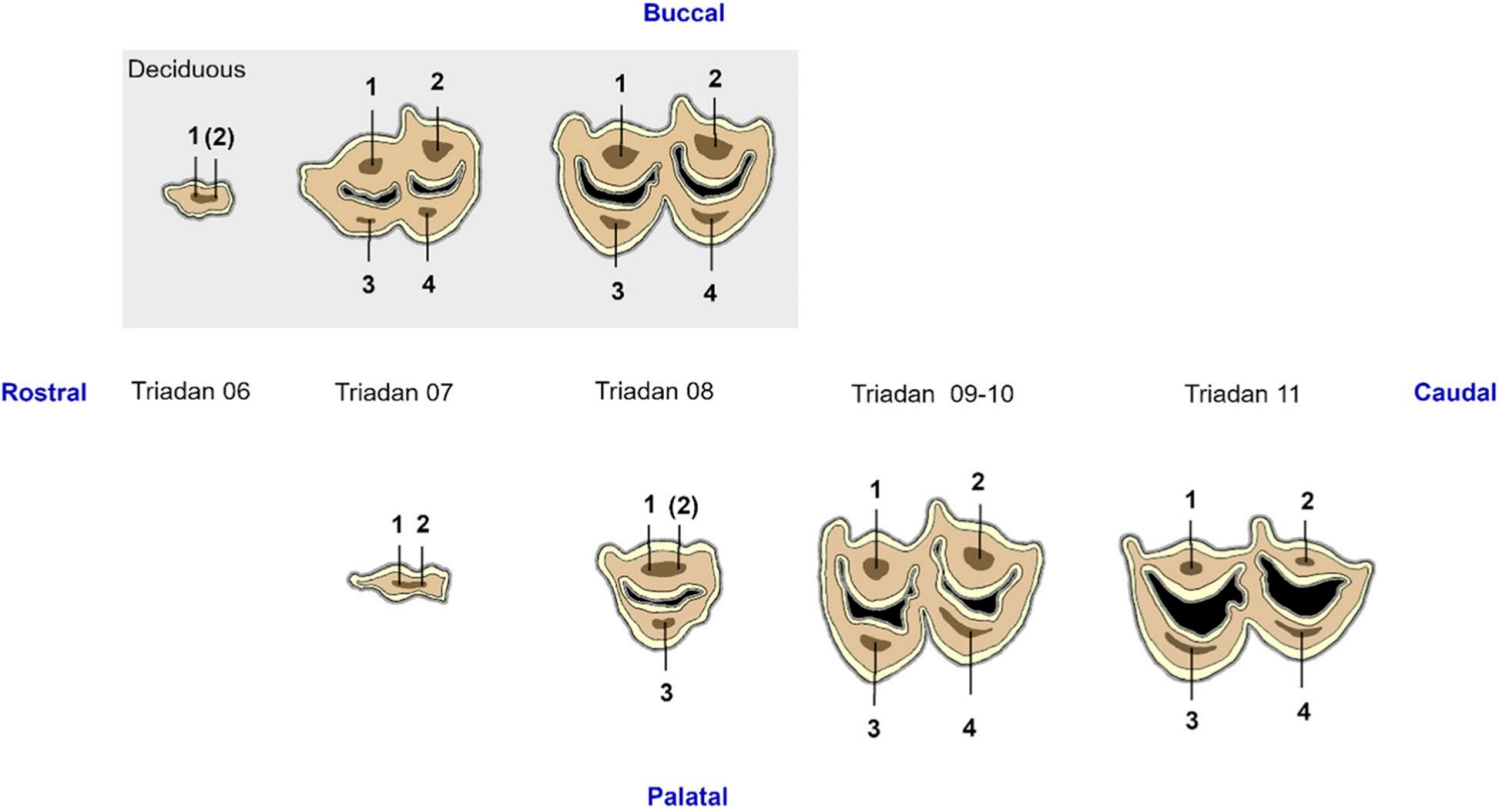
Pulp numbering system in alpaca maxillary cheek teeth. Pulp numbering system adapted from the equid numbering system by Du Toit et al. (2008). Deciduous teeth are displayed in a grey box. The occlusal surface consists of cement (dark grey), enamel (light yellow), dentin (light brown), darker secondary dentin overlying pulp horns (dark brown) and the lumina of the infundibula (black). Different pulp horns are numbered with Arabic numerals (1 to 4)

Apical deltas were defined as patterns of small accessory canals and small apical foramina at the apical region of well developed root structures. A previously described equine endodontic numbering system was used as a basis for the development of a pulp numbering system for identification of specific pulp horns in alpacas (Fig. 3) [8].

Different pulp configurations were recognised. A common pulp chamber (CPC) was defined as a pulp system in which communication existed between all identified pulp horns and root canals within the same tooth [5]. Pulpal segmentation created individual pulp entities lacking communication with neighboring compartments within the same tooth. Columnar segmentation (CS), as previously reported in mandibular cheek teeth, can be defined as the development of individual pulp entities caused by the disappearance of connecting intercolumnar pulpal tissue [8]. Additionally, within-column segmentation (WCS) was defined as a loss of communication between buccal and palatal aspects of the pulp system within the same column. Given the more complex nature of the pulp system in maxillary cheek teeth, both full and partial segmentation can be differentiated when studying both CS and WCS. Maximal segmentation (MS) was acquired in those teeth in which each pulp horn solely communicated with one root canal [5]. Measurements of SODT at the individual pulp horn levels were recorded by measuring the distance between the most occlusal aspect of a specific pulp horn and the most occlusal point of the overlying dentin [8].

### Statistical analysis

**Fig. 4:**
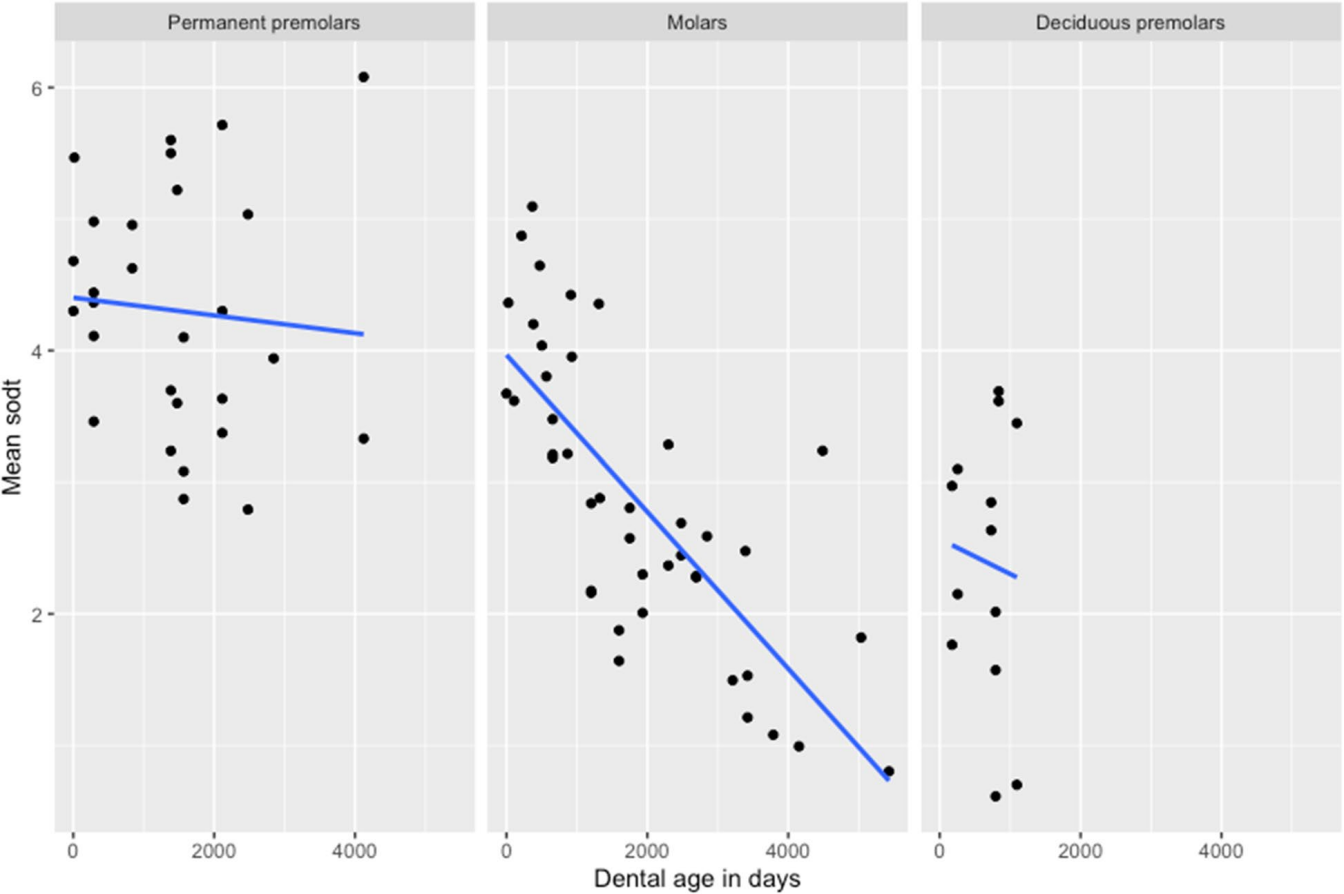
Scatterplots of the mean sub-occlusal dentinal thickness (SODT) in function of dental age. Scatterplots of the mean sub-occlusal dentinal thickness (SODT) in function of dental age (days) for the different types of dentition. The interaction between fixed effects ‘Dental age’ and ‘Type of dentition’ is apparent. Mean SODT decreases over time with differing slopes dependent on type of dentition, and tested statistically significant at the level of the maxillary molars (*P <* 0.001). No statistical analyses have been performed for the premolar cheek teeth given the relatively small sample sizes

Statistical analysis was reserved for the results of the SODT measurements. First, a descriptive analysis was performed on the SODT of all included cheek teeth. To take morphological differences at different Triadan positions into account, mean SODT of all included maxillary cheek teeth was calculated as a first variable of interest. A linear mixed model (lmer) was constructed with ‘individual alpaca’ added as random factor. Multiple fixed effects, comprising ‘Type of dentition’, ‘Dental age’, ‘Gender’ and ‘Mandibular arcade’ were evaluated univariably, as previously performed in mandibular cheek teeth [8]. Significantly associated factors ‘Type of dentition’ and ‘Dental age’ showed a strong interaction (Fig. 4). A split of the dataset based on type of dentition was required to allow further statistical analysis.

New univariable analysis were performed for all aforementioned fixed effects with mean SODT at the level of the molars specifically as variable of interest. No separate statistical models were build for the mean SODT at the level of the permanent and deciduous premolar cheek teeth given the low number of observations. On the other hand, the SODT above specific pulp horns in the maxillary molars was used additionally as a variable of interest. Aforementioned fixed effects were evaluated univariably, with ‘Triadan position’ nested within ‘individual alpaca’ added as random factors. Pulp horn was added as additional fixed effect in the latter model. After univariable analysis of aforementioned fixed effects for all variables of interest, factors with a *P <* 0.20 were retained for model building in the multivariable models. Associations between significant predictors were tested. Model building was performed backwards in a stepwise fashion, gradually excluding non-significant variables. Biologically relevant interactions between significant main effects were tested. Satterthwaite’s degrees of freedom method was used to obtain the *P*-values in each of the models. Normality was confirmed using a QQ-plot and the Shapiro-Wilk test on the residuals. To assess linearity, residuals were plotted versus fitted values. Residual plots were constructed to confirm the absence of homoscedasticity and potential outliers. Statistical significance was set at *P* < 0.05. Statistics were carried out using R V3.5.2., R Foundation for Statistical Analysis.

## Results

### General anatomy

**Fig. 5:**
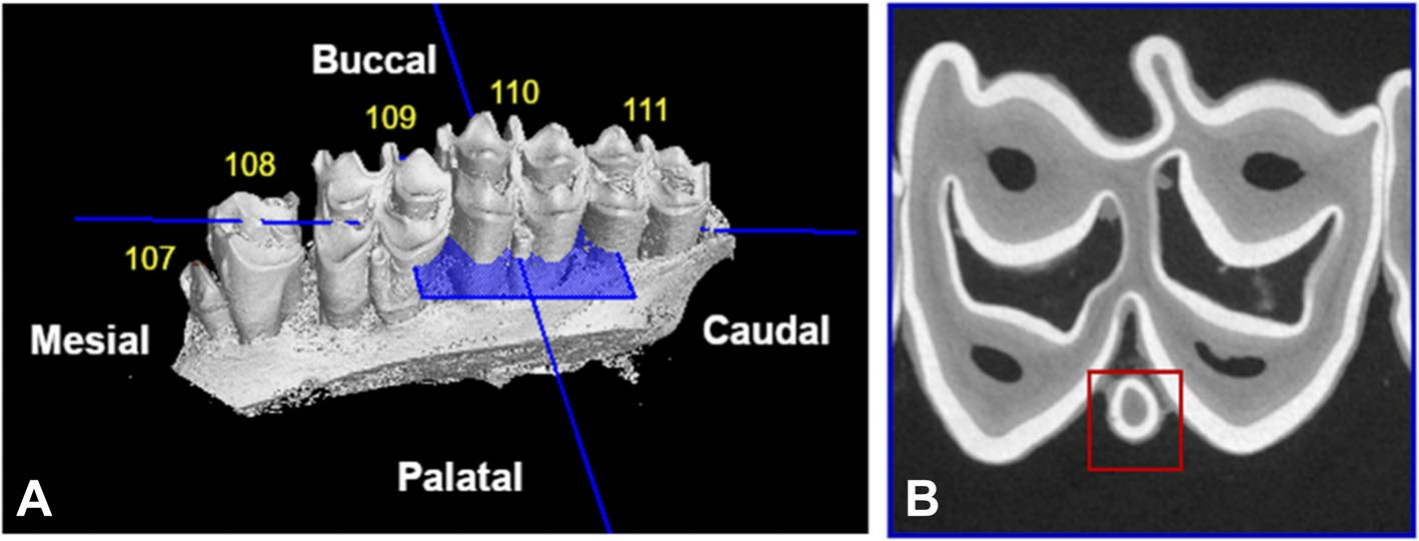
Alpaca pearl. Three-dimensional visualization of well-developed ‘alpaca pearls’ identified at the level of Triadan 109 and 110 in an 8-year-old alpaca (A). A rudimentary pearl is discernible at the level of Triadan 111. Note the open diastema between Triadan 108 and 109. B. A two-dimensional transverse slice of Triadan 110 ilustrates the alpaca pearl (red square)

Each maxillary arcade contained 3 to 6 cheek teeth which showed morphological differences. A small deciduous Triadan 06, consisting of a single column with 2 small roots, was found in the maxillary arcade of an animal aged 2 years and 2 months. Deciduous 07s and 08s were seen in maxillary arcades of all animals aged up to 3 years. Deciduous Triadan 07s showed a characteristic triangular shape at the level of the occlusal surface and consisted of 2 columns, 2 infundibula, 1 mesial root and 2 distal roots. Deciduous 08s showed a more rectangular appearance at the level of the occlusal surface and comprised an extra root at the mesial aspect in comparison to deciduous 07s. Each column contained one infundibulum and was connected to 2 roots. In the permanent dentition, only a small Triadan 07 could be perceived in animals aged 4 years and 3 months and older, which consisted of 1 column, no infundibula and two roots. A permanent 08 was present in all maxillary arcades of animals older than 3 years, and always consisted of 1 column, 1 infundibulum, 1 palatal root and 2 buccal roots. All three maxillary molars showed the same morphological characteristics of 2 columns, 2 infundibula and 4 roots. Each column included 1 infundibulum and 2 roots. Triadan 09, 10 and 11 were present in 95.7% (22/23), 87.0% (20/23) and 73.9% (17/23) of investigated maxillary arcades, respectively. Five maxillary molars from the same 8-years-old animal showed localized adherent dental tissue which the authors termed ‘alpaca pearls’. In all cases it was located at the level of the palatal invagination of the enamel, in-between two columns (Fig. 5).

### Number of pulp horns and pulp anatomy

**Fig. 6:**
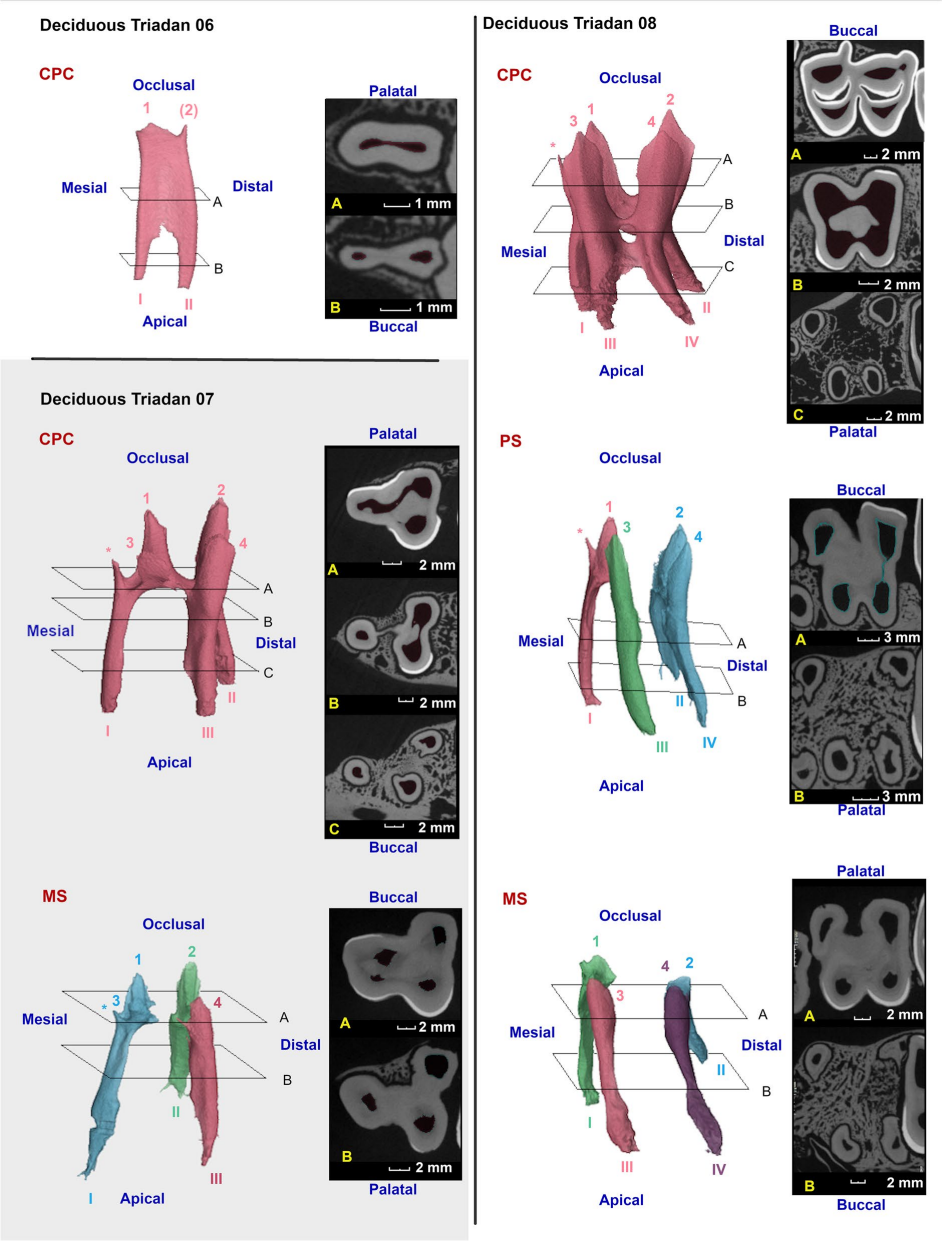
Three-dimensional reconstructions of recorded pulp configurations in deciduous maxillary cheek teeth in alpacas. Separate pulp compartments can be differentiated by different colours. Pulp horns and root canals are numbered by Arabic numerals ranging from 1 to 4, and Roman numerals ranging from I to IV, respectively. Additional occlusal pulp extensions, only found in younger cheek teeth, are indicated by an asterix (*). Inserted reference planes (A to C) indicate the position of added 2-D μ-CT images. Only one deciduous Triadan 06 (dental age of 2 years and 2 months) was included in the present study, which showed a common pulp chamber (CPC). Deciduous Triadan 07s showed two different pulp configurations. A CPC was detected as demonstrated in an 8-months old tooth. Furthermore, maximal segmentation (MS) was found as shown in a 2 years and 4 months old tooth. Pulp horns 1 and 3 remain connected to root canal I, whereas pulp horns 2 and 4 connect to root canals II and III, respectively. On the other hand, 4 root canals were identified in deciduous Triadan 08s leading to 3 differing configurations. A CPC is shown in a 6-month-old tooth. All 4 pulp horns and 4 root canals are connected. A 2 years and 4 months old tooth demonstrates partial segmentation (PS) with loss of columnar communication and within-column communication in the mesial column, creating 3 separate pulp compartments. In this specific case, communication exists between pulp horn 1 and root canal I, pulp horn 3 and root canal III and between pulp horn 2, 4 and root canal II and IV. A 3-year-old tooth demonstrates 4 separate pulp compartments, creating MS

**Fig. 7:**
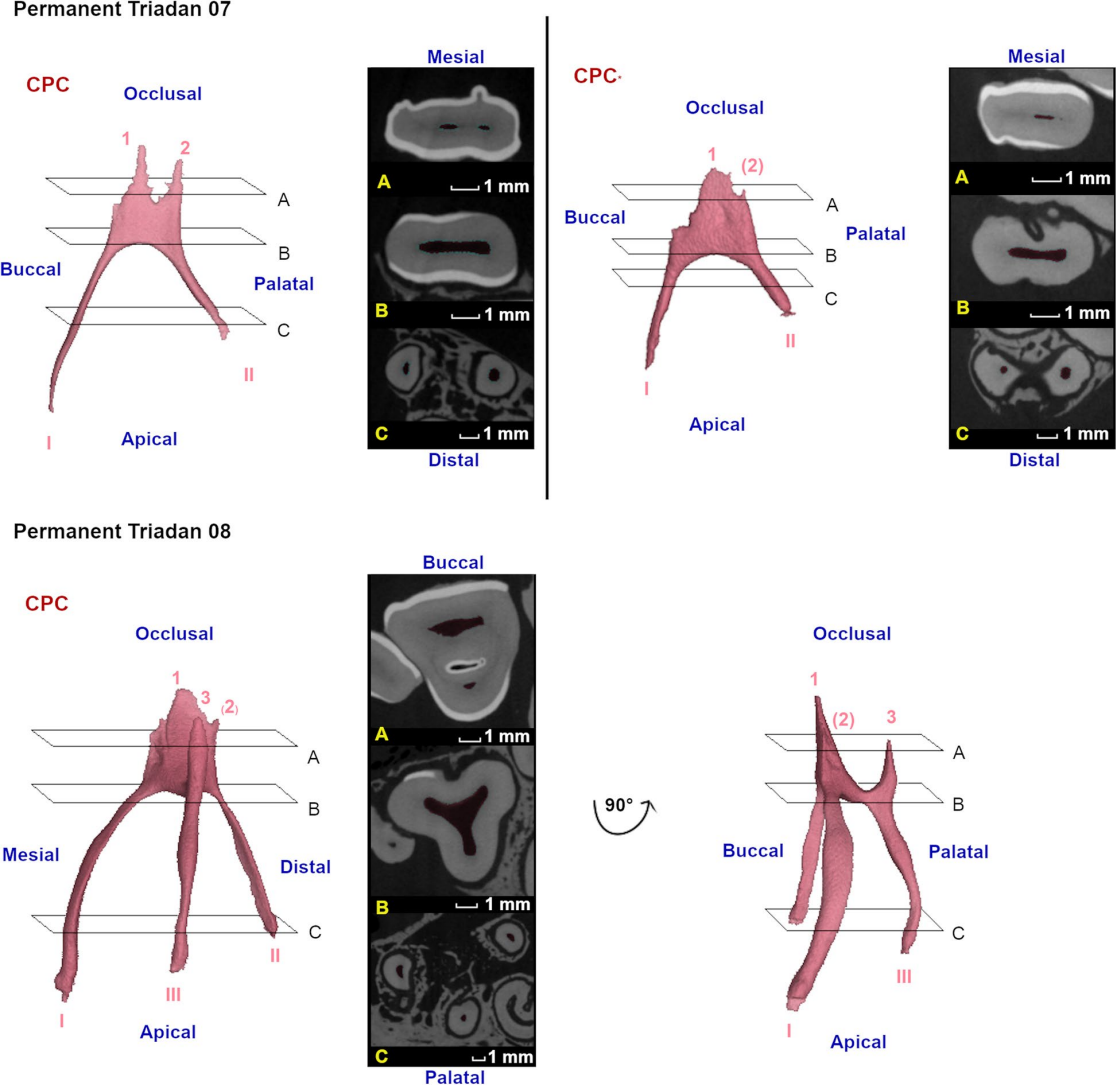
Three-dimensional reconstructions of observed pulp configurations in permanent maxillary premolars. Pulp horns and root canals are labeled with Arabic numerals ranging from 1 to 3 and Roman numerals ranging from I to III, respectively. Reference planes (A to C) indicate the position of selected 2D-images. A common pulp chamber was found in the majority of cases as demonstrated using the 3-D reconstruction of the pulp system of a 10 months-old *permanent Triadan 07* (CPC). In a 3 years and 10 months old tooth, occlusal pulp horn 2 is nearly absent. Also, shortening of the root canals occurs in older teeth (CPC*). At the level of *permanent Triadan 08 s*, a common pulp chamber was observed in all cases as demonstrated in a 7 years and 10 months old tooth. Only a rudimentary occlusal pulp horn 2 could be distinguished in some teeth

**Fig. 8:**
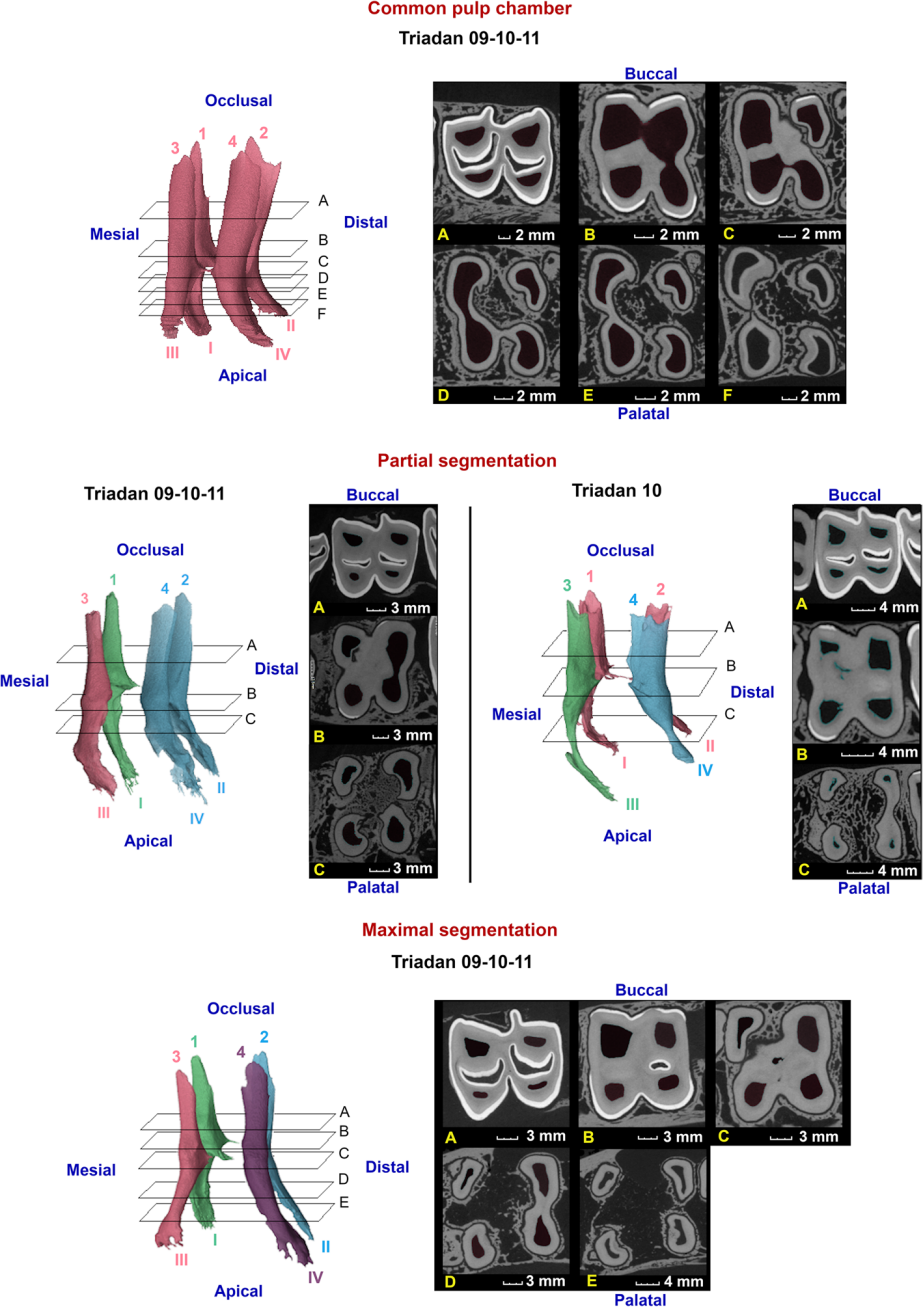
Three-dimensional reconstructions of the observed pulp configurations in maxillary molars. Pulp horns and root canals are labeled with Arabic numerals ranging from 1 to 4 and Roman numerals ranging from I to IV, respectively. Reference planes (A to F) indicate the position of selected 2D-images. Given the strong morphological similarities between molars at different Triadan positions, communal reconstructions have been constructed to illustrate different pulp configurations. A common pulp chamber is demonstrated in a 1 year and 5 months old Triadan 209, in which all pulp horns and root canals communicate. Two types of partial segmentation could be observed in other teeth. A first, as demonstrated in a 3 years and 7 months old Triadan 109, was found at the level of Triadan 09, 10 and 11 s. Communication existed between pulp horn 1 and root canal I, pulp horn 3 and root canal III and between pulp horn 2, 4 and root canal II and IV. An additional configuration was only found in one Triadan 10 (9 years and 4 months) characterized by 3 separate pulp compartments with a remaining mesio-distal communication between pulp horns 1 and 2. Compartments thus consisted of pulp horn 3 and root canal III, pulp horn 4 and root canal IV and pulp horn 1, 2 and root canal I and II. Maximal segmentation was observed at all Triadan positions in relatively older cheek teeth as demonstrated in a 3 years and 4 months old Triadan 210. Note the divergent direction of the reconstructed root canals observed in these specific teeth

Marked variations in pulpal anatomy between different maxillary cheek teeth were observed (Figs. 6-8). Two rudimentary pulp horns were identified in the single available deciduous 06, whereas all deciduous 07s and 08s contained four pulp horns (Fig. 6). A maximum of 2 rudimentary pulp horns were also observed in permanent 07s, whereas permanent 08s contained 1 palatal and 1 or 2 buccal pulp horns, the latter often splitting more occlusally. Four pulp horns were found in each of the studied maxillary molars (Triadan 09–11). Root length varied in relation to dental age. A wider apical foramen and shorter roots could be perceived in younger teeth. Variation in the presence and extent of apical deltas was observed in older cheek teeth. Freshly erupted teeth consisted of a simple straight conformation of the pulp system lacking root canals. With increasing age, root canals were observed to ramify and diverge creating apical deltas.

A CPC was present in all younger cheek teeth. Dependent on specific Triadan position, segmentation of the pulp system into separate pulp entities could be perceived at different dental ages. A CPC was demonstrated in 46/83 (55.4%) maxillary cheek teeth with a mean dental age of 2 years and 7 months (± 2 years and 5 months). Full CS was identified in teeth with a mean dental age of 6 years and 1 month (± 3 years and 6 months) (47.8%, 33/69). WCS was observed in 36/83 examined teeth with a mean dental age of 6 years and 4 months (± 3 years and 7 months). MS was identified in 31.3% (26/83) of examined cheek teeth with a mean dental age of 6 years and 8 months (± 3 years and 7 months). Detailed characteristics of CPC and pulpal segmentation can be found in Table 2.

**Table 2:** Schematic representation of the prevalence of different categories of pulpal segmentation in maxillary cheek teeth

	Triadan position							
	Deciduous			Permanent				
	6	7	8	7	8	9	10	11
% CPC	100%	67%	67%	85%	100%	31%	29%	25%
Oldest persistence of CPC	2y2m	2ym	2y2m	6y10m	11y4m	2y5m	2y6m	1y10m
Youngest loss of CPC	/	2y4m	2y4m	4y4m	/	3y7m	2y7m	1y10m
% CS (Full + Partial)	/	33%	33%	/	0%	69%	71%	75%
% Full CS	/	33%	33%	/	0%	69%	64%	75%
Oldest tooth without full CS	/	2y2m	2y2m	/	11y4m	2y5m	9y4m	1y10m
Youngest tooth with full CS	/	2y4m	2y4m	/	/	3y7m	2y7m	1y10m
% WCS (Full+Partial)	0%	33%	33%	15%	0%	69%	71%	75%
% Full WCS	0%	33%	17%	15%	0%	63%	50%	42%
Oldest tooth without full WCS	2y2m	2y2m	2y4m	6y10m	11y4m	3y7m	6y10m	12y4m
Youngest tooth with full WCS	/	2y4m	3y	4y4m	/	3y8m	3y4m	1y10m
% MS	0%	33%	17%	15%	0%	63%	43%	42%
Oldest tooth without MS	2y2m	2y2m	2y4m	6y10m	11y4m	3y7m	9y4m	12y4m
Oldest tooth without MS	/	2y4m	3y	4y4m	/	3y8m	3y4m	1y10m
Number of pulp compartments								
1	100%	67%	67%	85%	100%	31%	29%	25%
2	0	0	0	15%	0	6%	0	0
3	/	33%	17%	/	0	0	29%	33%
4	/	/	17%	/	/	63%	43%	42%

Dental age is expressed in years (y), months (m) and days (d). Categories include a common pulp chamber (CPC), columnar segmentation (CS), within-column segmentation (WCS) and maximal segmentation (MS). Given the clinical relevance with regards to endodontic procedures, an additional differentiation is made between full and partial segmentation for CS and WCS, with partial segmentation illustrating the situation in which communication is lost at 1 of the 2 possible connection sites

A CPC was found in the single studied deciduous Triadan 06 which showed communication between its two pulp horns and two root canals (Fig. 6). Deciduous 07s and 08s contained a CPC in 4/6 cases with a maximal dental age of 2 years and 2 months. All, older deciduous 07s showed 3 separate pulp compartments (Fig. 6). Pulp horns 1 and 3 both remained connected with mesial root canal I. In deciduous 08s, 3 or 4 separate pulp compartments were found in teeth with a dental age of 2 years and 3 months, and 3 years respectively. The first pulp conformation was characterized by communication between pulp horns 2 and 4, and root canals II and IV. The second configuration showed maximal pulpal segmentation with all pulp horns only communicating with a single root canal (Fig. 6).

Eleven of 13 studied permanent 07s showed a CPC. The central pulpal region was located relatively close to the occlusal surface. Often, only rudimentary pulp horns could be observed (Fig. 7). Obliteration of the distal aspect of the pulp system was found in an 11 years and 4 months old tooth. In a 4 years and 4 months old tooth, communication between the central pulp region and the mesial root canal was lost. A CPC was found in all studied permanent 08s. Maxillary molar pulp configurations (Triadan 09–11) showed strong similarities. Mesio-distal communication was lost in Triadan 09s, 10s and 11s in 62.5% (10/16), 28.6% (4/14) and 25% (3/12) of studied molars, respectively. No WCS was found without any CS. Details and age indications with regards to pulp segmentation at the level of specific Triadan positions are summarised in Table 2. Three different pulp configurations were found in Triadan 09s, including a CPC, full mesio-distal segmentation creating 2 pulp compartments, and MS creating 4 pulp compartments (Fig. 8). Similar to Triadan 09s, a CPC and MS were found in Triadan 10s. Additionally, 2 conformations with 3 separate pulp compartments were found in which full mesio-distal segmentation and partial WCS existed. Finally, Triadan 11s also showed a CPC and MS. Only one additional pulp conformation was found in which mesio-distal communication and within-column communication in the mesial column was lost.

### Physical contact between roots of adjacent cheek teeth

In newly erupting maxillary cheek teeth, roots appeared as rudimentary apical structures. With increasing age, lengthening and deviation in a mesial, distal, buccal or palatal direction occured. Adjacent cheek teeth were observed to have roots in contact with each other in 68.8% of cases. These only included animals aged 2 years and older, and were predominantly detected in Triadan 10s. Given the complex root morphology in maxillary cheek teeth, great variation was found in contact patterns between the different roots of adjacent teeth. The specific prevalence of physical root contact is illustrated in Fig. 2. These close contacts between adjacent roots have led to varying degrees of morphological adaptations of root tips in the majority of studied teeth.

### Sub-occlusal dentinal thickness

The SODT in maxillary cheek teeth was measured in 13 deciduous premolar, 28 permanent premolar and 42 molar cheek teeth, resulting in 47, 66 and 168 pulp horn SODT measurements, respectively. In general, SODT varied from 0.46 mm at pulp horn 2 in a 14-years-11-months-old Triadan 09, to 7.49 mm at pulp horn 1 in a 10-months-old permanent 08. Given the large structural differences between the different types of teeth, mean SODT at cheek tooth level was calculated to allow further comparative analysis. Mean SODT’s ranged from 0.61 to 3.69 mm (mean of 2.39 ± 1.03 mm) in deciduous premolars, from 2.79 to 6.08 mm (mean of 4.20 ± 0.93 mm) in permanent premolars, and from 0.80 to 5.10 mm (mean of 2.86 ± 1.11 mm) in molars (Fig. 4). Statistically significant differences in mean SODT were present between all types of dentition.

Multivariable analyses of the mean SODT at the level of the molars specifically, shows a statistically significant difference between Triadan positions 09 (2.50 ± 1.26 mm) and 10 (3.13 ± 1.01 mm) (*P* = 0.02). Furthermore, only a trend could be observed between mean SODT at the level of Triadan positions 09 and 11 (3.01 ± 0.97 mm) (*P* = 0.05). The mean SODT at the level of the maxillary molars was strongly associated with dental age (*P* < 0.001). Mean SODT decreased with increasing dental age (Fig. 4). With every increase in dental age with 1 year, the mean SODT at the level of a specific maxillary molar is expected to decrease with 0.24 mm (95%CI 0.17–0.31 mm). No statistical differences in mean SODT were found between female (2.69 ± 1.22 mm), male (3.07 ± 0.79 mm) and male castrated (3.47 ± 0.61 mm) animals. Also, statistical evidence for differing mean SODT values between the right (3.07 ± 1.20 mm) and left (2.65 ± 1.01 mm) maxillary arcade was lacking.

Maxillary molars showed strong morphological similarities allowing statistical analysis at pulp horn level for these teeth specifically. The SODT at the level of pulp horn 1 (2.46 ± 1.10 mm) was significantly lower in comparison to pulp horns 2 (2.96 ± 1.29 mm) (*P* < 0.001), 3 (2.81 ± 1.11 mm) (*P* = 0.001) and 4 (3.21 ± 1.25 mm) (*P* < 0.001). Furthermore, a statistically significant higher SODT at the level of pulp horn 4 could be detected compared to pulp horns 2 (*P* = 0.018) and 3 (*P* = 0.0002). SODT at specific pulp horns showed again a statistically significant decrease with increasing dental age (*P* < 0.001).

## Discussion

The aim of this study was to obtain novel insights into the (pulpal) anatomy of maxillary cheek teeth in alpacas as a prerequisite for the development of advanced tooth sparing treatment strategies. We have performed μ-CT analysis of maxillary cheek teeth of varying dental age. Detailed three-dimensional reconstructions and two-dimensional images with a resolution of approximately 0.05 mm were acquired for visualization and specific measurements.

Despite the higher efficiency in camelids, digestive strategies of both ruminants and camelids are similar and require fermentation of coarse forages in separate stomach compartments. Initial chewing aims at mixing the feed with saliva to form a bolus, which can then be swallowed. Feed is regurgitated and rechewed in both species. In camelids, chewing is performed with the mandible describing a figure-eight movement, thus including both horizontal and vertical movements. Whereas bovines perform mastication with a unilateral elliptical movement of the jaw [17]**.** Notwithstanding these differences in chewing pattern, cheek tooth anatomy in alpacas appears similar to the anatomy seen in ruminants. The selenodont occlusal surface appears to be well suited for the cutting and grinding of coarse feedstuffs in both chewing patterns.

Different cheek teeth showed important morphological differences [8]. Deciduous 07s showed a larger and more complex structure which consisted of 2 columns, 2 infundibula and 3 roots, whereas their permanent successors only comprised of one small column and two roots. Accordingly, deciduous Triadan 08s were taller with 2 columns, 2 infundibula and 4 roots. Their permanent successors only consisted of 1 column, 1 infundibulum and 3 roots. These marked differences could be helpful for the age determination of alpacas up to the age of approximately 3 years [13]. Other parameters that could help age estimation such as the previously described ‘llama buttress’ at the level of the mandibular cheek teeth could not be found in maxillary teeth [8]. A comparable structure located at the level of the palatal invagination of several maxillary teeth was observed in one animal and called ‘alpaca pearl’. The authors have noticed this structure only incidentally in other clinical patients. The very limited prevalence of this parameter excludes it from general use in relation to age estimation based on dentition for now. No other papers have reported on the existence of this structure.

Pulpal anatomy has been extensively studied and described in human dentistry and to a lesser extent in equine dentistry [5, 18–20]. The existence of a CPC in young alpaca cheek teeth has also been reported previously. Continuous deposition of secondary dentin at the periphery of the pulp chamber leads to narrowing and eventual CS [2, 8]. As previously reported in horses, the present results illustrate important variations in pulp configurations of alpaca maxillary cheek teeth [5]. It also identified the existence of both CS and WCS in maxillary cheek teeth, responsible for creating up to 4 separate pulp compartments within the same tooth. The youngest tooth with MS was a Triadan 11, aged 1 year and 10 months. The oldest tooth with maximal communication between all pulp horns was a Triadan 10, aged 2 years and 6 months. Knowledge of the pulp configuration is of primordial importance when dealing with dental disease. The present study provides insights into the timeframe at which separate pulp compartments develop in specific maxillary cheek teeth. As pulp disease can remain confined to such separated parts of the tooth’s pulp system, the degree of pulpal segmentation is of particular interest in the decision making process regarding the use of tooth saving techniques.

With increasing age, maxillary cheek teeth showed a varying degree of root divergence. Tooth sectioning was previously described as an aid to facilitate oral extraction of sectioned mandibular cheek teeth under differing angles [8]. In the authors’ clinical experience, oral extraction of non-sectioned maxillary molars is practically feasible in all but rare cases. Maxillary roots seem relatively short in relation to the crown and demonstrate a limited degree of divergence when compared to mandibular roots, especially in relatively young maxillary cheek teeth. Sectioning of maxillary cheek teeth prior to extraction should therefore only be considered in older teeth, with extensive root development and pronounced deviation of the root tips, and thus increased risk of root (tip) fracture. Several complicating factors should be taken into account when considering sectioning of maxillary versus mandibular cheek teeth. These include the presence of 4 instead of 2 diverging roots and the limited mid-tooth buccal enamel invagination, creating a thicker bridge to section. Moreover, adjacent columns within a single cheek tooth appear to be connected over a rather long occlusal-apical distance in comparison to the situation in mandibular cheek teeth, rendering tooth sectioning technically more demanding, however not impossible. A loss of integrity of created tooth segments can be expected when performing both column and within-column sectioning to address differing extraction angles for the 4 diverging roots. In a similar fashion, single-rooted crown-root segments have been demonstrated to be prone to fracture when extensive levering forces are exerted during tooth extraction in cats [21–23]. Therefore, only column segmentation can be advised in specific cases.

Focal dental disease limited to one column of a specific tooth has been reported in alpacas, theoretically allowing tooth sectioning at the column level followed by partial extraction of only the affected part in the concerning cheek tooth [1]. However, no scientific histological evidence exists conforming disease only to be confined to one column and respective root(s) without any interference with the normal histological appearance of the respective other tooth half. Nevertheless, aforementioned technique remains rather troublesome in maxillary cheek teeth comparison to performing the same procedure in mandibular cheek teeth [8]. Aforementioned limitations concerning tooth splitting remain as important, highlighting the difficulty level and associated low success rate of performing the ‘perfect sectioning’ required in this specific situation. Nevertheless, novel insights into possible pulp configurations and more specifically, communications inbetween pulp tissue of different columns within the same tooth are provided for the potential further development of this specific technique in the future (Table 2). The dental age of full CS is of primary interest to justify the use of this specific technique and showed wider variation in comparison to mandibular cheek teeth. Full CS was seen between 2 and 4, 2.5 and 9.5, and around the dental age of 2 years in Triadan 09s, 10s and 11s, respectively.

Given the low number of cheek teeth contributing to the occlusal surface, tooth saving strategies are of primary importance in alpacas. When dental disease and more specifically, pulpitis is diagnosed in an early stage, specific endodontic procedures can be an important way to save diseased teeth and thus prove worthy to be explored in this species. Prior attempts to perform retrograde endodontics following apicoectomy have proven to be unsuccessful, most likely due to a lack of proper knowledge of the pulp system in the alpaca, as was also reported in early endodontic development in human and equine dentistry [6, 24–26]. The present study results can provide a basis for the development of endodontic techniques as it provides a better insight into possible communications between different pulp horns and root canals (Table 2). However, a large variation in pulpal communications could be found, including WCS, which couldn’t be detected at the mandibular level [8]. The oldest teeth in which some kind of communication between 2 of 4 possible pulp segments persisted were of dental ages 3.5, 9.3 and 12.3 years in Triadan 09, 10 and 11, respectively. Given the large variation in the degree of communication between the different pulp segments, computed tomography (CT)-scan analysis of affected teeth is recommended prior to attempting any tooth saving techniques on cheek teeth in the maxillary arcade. Nevertheless, resolution differences between μ-CT scans and standard CT-scans performed in a clinical setting should be kept in mind. Resolution limitations of the latter technique could be responsible for missing small interpulpal communications, which may be identified on micro-CT scan images in the present study. Specific studies comparing both imaging techniques in alpaca cheek teeth are of interest to substantiate this hypothesis. Utilising a significantly lower radiation dose, cone beam CT is increasingly used in human (and small animal) dentistry as a usefull diagnostic and evaluation tool of the internal endodontic root canal system prior to performing endodontic treatments [27, 28]. Cone beam CT has been proven to be superior to conventional CT when imaging the dentition in rabbits by providing higher quality images [29]. However, the usability and value of this technique in alpacas still needs to be validated. Meticulous case selection, primarily excluding cheek teeth with a severely compromised periodontal region is of utmost importance for the success of any endodontic procedure. Clinical research providing a long-term follow-up of endodontically treated cheek teeth can shed light on the value of these specific advanced techniques in alpaca cheek teeth. Space limitations caused by the combination of a relatively large tongue and the pronounced rostral location of the lip commisures complicate all intraoral procedures by limiting the range of motion for manipulation of instruments [3].

Physical contact of adjacent roots resulting in deformation, shortening and partial destruction of cheek teeth root tips has recently been suggested as a risk factor for dental disease based on a CT-study of healthy dental structures in new world camelids [2]. Despite the clear presence of root tip deviation, deformation and shortening resulting in morphological adaptations, evidence of destructive changes could not be found based on the analysis of the acquired μ-CT images in the present study. Previoulsy, physical contacts between root tips of adjacent maxillary cheek teeth was reported in a minority of cases [2]. Surprisingly, a very high prevalence of these physical contacts was found in the present study. Root tip deviation and subsequent morphological adaptations including deformation and shortening were detected at the level of adjacent roots IV/III and II /I in 82 to 94% of apical zones. The close proximity of adjacent root tips might be responsible for impeding normal root development and could subsequently induce morphological adaptations accompanied by an inflammatory response of surrounding tissues. It can be hypothesized that this might render these specific apical zones more susceptible to bacterial colonization and thus development of anachoretic infection. Additionally, these changes could possibly act as facilitating factors for the spread of infection, affecting multiple maxillary cheek teeth as is often observed in chronically diseased cases. However, additional histological research of the apical region in maxillary and mandibular cheek teeth is required to examine the pathophysiological effects of these physical contacts and their relation to dental disease in alpacas.

Scientific literature on dental disease in alpacas reports a significantly higher prevalence of tooth root abscesses, recently termed ‘apical infections’, at the level of the mandibular cheek teeth [1, 24, 30, 31]. Mandibular apical infection is often readily palpable or sometimes even visible whereas a similar swelling at the level of the maxillary arcade is frequently absent in alpacas [3]. During the selection procedure for the present study and after general interpretation of acquired μ-CT images, 9.4% (3/32) of studied permanent premolars and 30.5% of studied molars were excluded for further analysis of normal pulp morphology and measurements of the SODT due to the presence of severe dental disease, primarily because of apical disease despite the absence of an external maxillary swelling. In most of these cases, multiple maxillary cheek teeth originating from the same dental arcade were excluded, illustrating the dessimination of the disease process. As maxillary tooth pathology is often only diagnosed accidentally when performing an oral examination in an animal presented with a mandibular swelling, it is of primary importance to pay specific attention to the maxillary arcades whenever performing an oral examination or using specific medical imaging modalities. Other non-specific clinical signs such as a decreased appetite, subsequent weight loss and decreased body condition score, quidding and hypersalivation are reported to be a sign of ongoing mandibular or maxillary dental problems [4]. Alpacas are notorious for their ability to hide clinical signs related to even advanced disease processes. Also the listed symptoms are easily overlooked which often leads to a late diagnosis of even severe dental disease in this species. In the authors’ experience, few alpacas have been presented with nasal discharge related to a maxillary cheek tooth infection and associated secondary sinusitis, as is more often seen in horses [32–34]. The introduction of routine oral examinations in the management of alpacas can be of particular value in the early detection of dental disease.

The production of sub-occlusal secondary dentin is a dynamic and multi-factorial process which counteracts the continuous wear caused by attrition and abrasion at the occlusal surface, preventing exposure of vital pulp tissue [16, 20, 35]. The regulation of this process seems at least partly regulated by the degree of wear-induced stimulation [35–37]. Tooth wear rates in camelids can vary strongly according to dietary, nutritional, health and other factors [13]. A higher mean SODT at the level of the permanent premolar cheek teeth was found in comparison to the other types of dentition, as also previously demonstrated in mandibular cheek teeth [8]. The relatively low decrease in SODT with increasing dental age may be indicative for a relatively low rate of attrition in permanent premolars (Fig. 3). However, a larger sample size of teeth would allow to draw more precise statistical conclusions. When considering the mean SODT of the maxillary molars, a significant decrease was detected with increasing age. The lower mean SODT in maxillary Triadan 09s specifically could be partly attributed to their relatively early eruption in comparison to the adjacent caudal molar cheek teeth. Disruption of the balance of sub-occlusal dentin production and wear may be responsible for this phenomenon. We hypothesize that odontoblasts in older cheek teeth may not be able to compensate for the continuous high rate of wear through a sufficient sub-occlusal dentin production during the complete lifetime of the animal. However, further research is necessary to explore this hypothesis given no data are available on this specific subject. A previously stated hypothesis indicates that larger masticatory forces could possibly be centered around the Triadan 09 position causing a higher wear rate at this specific position [3]. However, further research focusing on masticatory forces exerted at the occlusal surface and the general chewing cycle in this species remains necessary to draw any definite conclusions. The extremely low SODT value of 0.46 mm measured over pulp horn 2 in a 14 years and 11 months old Triadan 09 specifically demonstrates the significant risk of inadvertently causing pulp exposure when performing dental floating in older maxillary cheek teeth. A generally low SODT and wide variation should always be kept in mind when performing reductions in these species. Any crown reductions should be performed conservatively and with constant attention to the secondary dentin overlying pulp horns. Furthermore, care should be taken to extrapolate findings in domesticated animals in this study to animals housed in the original conditions.

This study is the first to perform μ-CT examinations to examine general anatomy and specific pulp morphology in maxillary alpaca cheek teeth. The cross-sectional study design is a limitation inherent to the utilized scanning method. Dose limitations in live animals render μ-CT examinations impossible, precluding a longitudinal study design. Also practical feasibility concerning optimal sample positioning in live animals is troublesome. Sufficient teeth within different age intervals were selected to counter this specific limitation and to gain clear insights into specific maxillary cheek tooth morphology. Selection bias cannot be ruled out since all samples were retrieved from the pathology department. Exclusion of the large portion of cheek teeth diagnosed with severe dental disease primarily consisting of apical disease prevented interference with the acquired study results in healthy teeth. Measured SODT values are expected not to be influenced by dental floating as this is not routinely performed in alpacas.

## Conclusions

The results of the present study are complementary to the results of a comparable study on mandibular cheek tooth anatomy in alpacas by the same authors. This is the first study to utilize advanced imaging techniques to gain a better understanding of maxillary cheek tooth morphology in alpacas. More specifically, pulp morphology, physical root contacts and SODT were of primary interest given their clinical significance. Thes results of this study provide novel insights into the morphological variation of the pulp which is a prerequisite for the development of endodontic techniques, primarily of interest when apical disease is diagnosed in an early stage. Morphological adaptations of cheek teeth roots are shown to be highly prevalent. Their role in the pathogenesis of apical disease warrants further investigation. A conservative approach is recommended when performing dental floating of alpaca maxillary cheek teeth, especially in older teeth to avoid inadvertent pulp exposure.

## Data Availability

The datasets generated and/or analyzed during the current study are not publicly available due to ongoing research projects but are available from the corresponding author on reasonable request.

## Abbreviations

CPC: Common pulp chamber
CS: Columnar segmentation
CT: Computed tomography
MS: Maximal segmentation
SODT: Sub-occlusal dentinal thickness
WCS: Within-column segmentation
μ- CT: Micro-computed tomography
